# Plea for More Artistic Work in Dental Prosthesia

**Published:** 1904-01

**Authors:** Roland D. Jarvis

**Affiliations:** London.


### PLEA FOR MORE ARTISTIC WORK IN DENTAL PROSTHESIA.

By Roland D. Jarvis, L. D. S., D. D. S., London.

Commencing from the moment a patient's wants are known, an artificial denture, say, for the upper jaw, I will describe my method for the successful making of said case from beginning to end If there are two or more teeth still remaining in the anterior portion of the jaw and which must be extracted, the first thing I do is to take what we call a labial expression of these remaining teeth and lower teeth as they then occlude. (The smae kind of an impression is taken in orthodontia work before and after completing a case of regulating.) While preparing for the extraction, I have been engaging the patient in conversation and laughter, if possible, so that I may note in my mind whether or not the gums are ever exposed to view, for if so, gum section teeth must invariably be used if you wish to turn out a satisfactory piece of work both to patient and yourself. I have not as yet come across a pink rubber that can imitate the color of the natural gums as closely as the porcelain. I am taking for granted the patient intends waiting till the gums and alveolus have receded sufficiently to allow the insertion of a permanent denture. Notes are made on the case and laid away with the labial impression; the patient is dismissed, and returns in due time for the impression.

The Impression.—Pick a tray the shape of which corresponds to that of the jaw, but it must not fit the gums too closely, the shape of the tray being of more importance than an accucrate fit. Into your mixing bowl pour some lukewarm water, using nothing else than the warmth of the water to hasten setting of plaster. Add plaster by degrees, allowing it to settle as put in till dry plaster appears over all the surface; et it stand a few moments, then mix thoroughly, the plaster now being of such a consistency that it will not run or drop from the cup; carry to position in the mouth, forcing heels of cup up first and separately before anterior portion of cup. When up in place, press cheeks and lips in towards the gums to force the paster well up into place. Take out when sufficiently hard to hold together, and run immediately; separate and form a compound plate to fit the model for bite.

The Bite.—This is something I hardly ever have any trouble in getting correctly. First, mentally note the distance the jaws are apart when the face is resting naturally, then place sufficient wax on the compound plate to a little more than fill the space between upper gums and lower teeth, and carry all to position against upper gums. Before having patients close on wax, have them sitting in an easy, *upright position*, and tell them to relax the muscles of the jaws, so that the face assumes its normal lines when resting easily; and, when satisfied as to this, the patient is directed to close *sl wly* far enough only to bring the jaws into the normal position. Too much wax on the compound plate is the main cause of so many inaccurate bites. When you have marked the lip line and median line of the lip on wax, take it out if hard.

The next step, an all-important one, is the selection of the teeth to be used; and, to aid you in this, you have the model run from the labial impression before referred to, and you must take into consideration the lower teeth and stature of patient for size and shape, and complexion for shade of teeth to be used. If single teeth are to be utilized, break up a half dozen sets if necessary, but do not send a denture out of the office the teeth on which are all the same shade. If you observe the natural teeth closely, you will see that the laterals are a shade lighter than the centrals, and the cuspids a shade darker, the bicuspids about the same shade as the centrals, and molars a trifle darker. In using and sticking to the teeth of one set only, there is too much of a sameness about the appearance when finished, a look of artificiality that is only too much apparent. Of course if gum-section teeth are to be used, you cannot be so particular as to shades as when single teeth are used.

A suggestion or two in reference to gum-section sets will perhaps not be amiss here. When using them use the Samson rubber in preference to any other, for the reason that it becomes more plastic under heat, and any surplus will escape more readily from the flask, lessening the danger of dark joints. Use the Donham spring press for the closing of flask *in the vulcanizer*, when the rubber reaches its most plastic conditon. V-shape the joints when grinding, and when flasked pack with cement and, if possible, let the case remain over night before packing, so that the plaster can harden well.

When a case is finished, you can grind and mutilate the teeth as much as you wish, only do not have them looking all alike and even when inserted.

General Remarks.—Show patients that you take an interest in them, advise them as to the probable difficulty they will experience in getting used to the plate, and to be sure and return if the plate is giving any pain. Do not let the patients leave the office thinking that now you have been paid for the work, you do not wish to see them again or care how they get along. Be absolutely honest with your patients, showing an interest in their progress in getting used to the denture, and your success in this branch of dentistry will be assured.—*D minion.*

